# Assessment of Dietary Soy Isoflavones Intake Among Women Aged 14 Years and Older in China: A Population-Based Cross-Sectional Study

**DOI:** 10.3390/nu18162696

**Published:** 2026-08-18

**Authors:** Qiuyan Shi, Fanglei Zhao, Feng Pan, Dechun Luan, Jianwen Li, Yuxia Ma

**Affiliations:** 1 School of Public Health, Hebei Medical University, Shijiazhuang 050017, China; qiuyanshi@stu.hebmu.edu.cn; 2 NHC Key Lab of Food Safety Risk Assessment, Research Unit of Food Safety, Chinese Academy of Medical Sciences (No. 2019RU014), China National Center for Food Safety Risk Assessment (CFSA), Beijing 100022, China; zhaofanglei@cfsa.net.cn (F.Z.); panfeng@126.com (F.P.); 3 Institute of Nutrition and Food Hygiene, Liaoning Provincial Center for Disease Control and Prevention, Shenyang, 110172, China; dechunluan@163.com

**Keywords:** soy isoflavones, dietary intake, food contribution rate, specific proposed level, Chinese women

## Abstract

**Objective**: This study assessed dietary soy isoflavone (SIF) intake among Chinese women ≥14 years, identified determinants, and evaluated adequacy against the Specific Proposed Level (SPL) in Chinese Dietary Reference Intakes, to inform targeted guidance. **Methods**: Intake was estimated using a distribution model combining 2017–2020 China National Food Consumption Survey data with SIF concentrations from food composition tables. Demographic differences and adequacy versus age-specific SPL were examined; supplemental doses were calculated for individuals below SPL. **Results**: Mean SIF intake was 14.77 mg/standard person-day, generally low. Significant differences (*p* < 0.05) were observed across most demographic subgroups except education. Higher mean intakes (mg/day) were seen in women aged ≥65 years (16.20), rural residents (16.38), light activity level (15.17), annual income > 100,000 CNY (15.51), manual laborers (16.11), and central China residents (highest, 21.01). Dried legumes and products, particularly tofu and its derivatives, contributed about 80% of total intake, with tofu-derived items accounting for 57.62–71.49%. Across all age groups, intake was significantly below the SPL, with >96% of participants failing to meet the recommendation. To reach the SPL, premenopausal women require ~40 mg/day supplemental SIF (equivalent to ~45 g soybeans/products), while perimenopausal and postmenopausal women require ~60 mg/day (~60 g soybeans/products). **Conclusions**: Dietary SIF intake among Chinese women ≥14 years is substantially below the SPL, a deficit that may attenuate associated health benefits. As dried beans and bean products are the dominant contributors, promoting these foods represents a feasible strategy to improve population-level SIF adequacy.

## 1. Introduction

Soy isoflavones (SIF) are polyphenolic flavonoid compounds produced as secondary metabolites of leguminous plants and are found in particularly high concentrations in soybeans. Within soybean seeds, SIFs are predominantly localized in the cotyledons and hypocotyls, with only trace amounts present in the seed coat [1]. Since their initial characterization in the early 20th century, 12 structurally distinct SIF compounds have been identified, occurring primarily as three aglycone forms and nine conjugated glycosides. Conjugated glycosides account for approximately 97–98% of the total SIF content, whereas the free aglycones—generally regarded as the biologically more active forms—constitute the remaining 2–3%.

Perimenopausal and postmenopausal women experience a progressive decline in estrogen secretion, which is associated with an increased risk of obesity, type 2 diabetes, osteoporosis, cardiovascular disease, and breast cancer [2,3,4,5,6]. Although estrogen replacement therapy (ERT) can alleviate these conditions and attenuate estrogen deficiency related bone loss, concerns regarding its potential adverse effects—including increased risks of cardiovascular events, breast cancer, and endometrial cancer, particularly in high risk high-risk populations such as cancer patients—have limited its broader use [7,8,9,10,11]. Therefore, safe and effective alternative strategies, especially dietary interventions, have attracted increasing research attention [12,13].

In this context, SIFs have emerged as a promising dietary strategy. The molecular structure of SIF closely resembles that of endogenous human 17 β estradiol, enabling these compounds to bind to estrogen receptors and act as selective estrogen receptor modulators (SERMs) [14,15,16,17,18]. Through this bidirectional regulation mechanism, SIFs may exert weak estrogenic effects in low estrogen environments while exhibiting potential antagonistic activity under high estrogen conditions, supporting their characterization as naturally occurring SERMs [19,20,21]. In addition to their estrogen like activity, SIFs possess well documented antioxidant properties, including inhibition of reactive oxygen species and hydrogen peroxide generation, attenuation of oxidative DNA damage, and suppression of lipid peroxidation, collectively reducing oxidative stress related stress-related cellular injury [22]. One study reported a reduction in oxidative DNA damage in healthy women following six months of SIF intervention [23].

The estrogen like activity of SIFs may contribute to the alleviation of menopausal syndrome and the amelioration of postmenopausal osteoporosis. Evidence indicates that SIF consumption reduces the frequency of hot flashes [24,25,26,27,28,29,30,31,32,33] and modulates serum follicle stimulating hormone (FSH) and luteinizing hormone (LH) levels, thereby improving menopausal symptoms. However, some studies have reported no significant improvement in vasomotor symptoms, including hot flashes and night sweats. Furthermore, SIFs and their principal metabolite equol may enhance osteoblast activity, promote bone matrix production and secretion, and facilitate bone mineralization, effects that may increase bone mineral density at the lumbar spine, hip, and femoral neck while also inhibiting bone resorption [34,35,36,37,38], and may help improve osteoporosis related outcomes in postmenopausal women [39,40,41].

Beyond menopausal and skeletal health, SIFs have been associated with additional potential health benefits. In relation to cardiovascular health, SIF is highly effective in lowering cholesterol and regulating blood lipids, with premenopausal women experiencing a greater reduction in cholesterol levels than postmenopausal women [42], SIFs may improve endothelium dependent vasodilation in healthy individuals and in those with hypertension, and may reduce arterial stiffness [43]. SIF supplementation has also been reported to lower total cholesterol levels in postmenopausal women [44,45] and in patients with type 2 diabetes. Furthermore, epidemiological evidence suggests that SIF intake is associated with a reduced risk of several cancers among Asian non-smoking women, including endometrial, colorectal, ovarian, and lung cancers [46,47].

Notably, while the most extensively documented benefits of SIF pertain to perimenopausal and postmenopausal women, the Chinese DRIs establish SPLs for all women aged 14 years and older. Given that 14 years corresponds to the average age of menarche in Chinese females—marking the onset of the reproductive lifecycle and endogenous estrogen exposure—we included this age threshold to comprehensively evaluate SIF intake across the entire reproductive continuum, from adolescence through postmenopause. This approach allows for the identification of inadequacies not only in midlife women but also in younger cohorts who may also fall short of the recommended levels.

Although SIF offers numerous health benefits for women, there is currently little research on SIF intake in China or elsewhere in Asia. This study aimed to systematically evaluate dietary SIF intake among premenopausal, perimenopausal, and postmenopausal women in China using a nationally representative food consumption data. We further quantified the discrepancy between actual SIF intake and the specific proposed level (SPL), with the aim of providing evidence to inform the development of precision nutrition intervention strategies for women with insufficient SIF intake within the existing food system.

## 2. Materials and Methods

### 2.1. Data Source

#### 2.1.1. Food Consumption Data

Dietary data were obtained from the nationally representative 2017–2020 Chinese Food Consumption Survey (CFCS), organized and conducted by the National Center for Food Safety Risk Assessment. A multistage stratified cluster random sampling method proportional to population size was employed. A total of 126 survey sites were selected across 23 provincial-level administrative divisions in eastern, central, western, and northeastern China, including Beijing, Fujian, Guangdong, Hebei, Jiangsu, Shandong, Shanghai, Tianjin, Zhejiang, Henan, Hubei, Hunan, Jiangxi, Gansu, Inner Mongolia, Guangxi, Guizhou, Shaanxi, Yunnan, Chongqing, Heilongjiang, Jilin, and Liaoning.

At each survey site, three subdistricts or townships were selected using probability proportional to size sampling. Within each subdistrict or township, four residential or village committees were randomly selected, and ten households were randomly chosen from each committee, yielding 120 households per survey site.

The CFCS (2017–2020) was approved by the Institutional Review Boards of the China National Center for Food Safety Risk Assessment (Protocol Number: No. 2025188). Written informed consent was obtained from all participants aged ≥3 years; for preschool children, written consent was provided by their parents or legal guardians.

After excluding 23,232 males, 3536 females aged younger than 14 years, 475 pregnant or lactating women, 48 participants with implausible energy intake values as >4000 kcal/day or <600 kcal/day, and one participant with missing demographic data, a total of 20,676 participants were included in the final analysis.

#### 2.1.2. Soy Isoflavone Content Data

The SIF concentration data were obtained from the sixth standard edition of the Chinese Food Composition Tables, which encompass 326 food entries. For foods not directly listed in the database, SIF concentrations were estimated by referring to botanically or compositionally similar foods. For example, the SIF content of certain chili pepper varieties was estimated using values for bell peppers given their same botanical family and genus.

### 2.2. Food Classification

A hierarchical food classification framework was applied. At the first tier, foods were classified into 11 groups: dried legumes and legume products; vegetables and vegetable products; condiments; ethnic foods and pastries; cereals and cereal products; fruits and fruit products; tubers, starches and products; nuts and seeds; fungi and algae; milk and milk products; and fast foods.

At the second tier, dried beans and bean products were further divided into 4 subcategories according to processing method and physical form: tofu and tofu-derived products, whole beans, soy-based beverages, and others.

### 2.3. Participant Stratification

Participants were stratified by age, urban or rural residence, physical activity level (PAL), annual household income, educational attainment, occupation, and geographic region. Because direct information on reproductive stage was unavailable in the CFCS, menopausal status was classified using an age threshold of 45 years, consistent with the Stages of Reproductive Aging Workshop (STRAW) criteria [48] and published data on age at natural menopausal in Chinese woman [49,50]. Women aged <45 years were classified as premenopausal, whereas those aged ≥45 years were classified as perimenopausal or postmenopausal.

### 2.4. Assessment of SIF Intake

SIF intake for each participant was calculated using a simple distribution model, according to the following formula:

(1) $$SIF\;intake=\sum_{n}^{i=1}\frac{F_{i}\times C_{i}}{100}$$

*SIF intake*: actual daily SIF intake per person, expressed as mg/d;
*F_i_*: daily consumption of food item i, expressed as grams per person per day, corresponding to the edible portion consumed as assessed using the 24-h dietary recall method;
*C_i_*: SIF concentration of food item i, expressed as mg/100 g;
100: Unit conversion coefficient;
*n*: the number of food items included in the calculation.

### 2.5. Standardization to Per-Standard-Person-Day Intake

To facilitate comparison of SIF intake across age groups and geographic regions, individual intake was standardized to a per-standard-person-day basis. A standard-person coefficient was calculated for each participant by dividing the individual estimated energy requirement (EER), determined according to age, sex, PAL, and physiological status, by the EER of a reference standard person. The reference standard person was defined as an 18-year-old male with light activity level and an EER of 2150 kcal/day, according to the Chinese DRIs. Standardized daily SIF intake was calculated as follows:

(2) $$Standard\;person\text{-}day\;SIF\;intake=\frac{SIF\;intake}{E_{1}/E_{2}}$$

- *Standard person-day SIF intake*: SIF intake per standard-day, expressed as mg/standard person-day;
- *E*_1_: the EER corresponding to an individual’s age, gender, physical activity level, and physiological condition, measured in kcal/day;
- *E*_2_: the EER of the reference standard person, defined as 2150 kcal/day.

### 2.6. Dietary Reference Values and Intake Targets

#### 2.6.1. Adequacy of SIF Intake

Adequacy of SIF intake was assessed against the SPLs specified in the 2023 edition of Chinese DRIs. The SPL is 55 mg/day for premenopausal women and 75 mg/day for perimenopausal and postmenopausal women. The Tolerable Upper Intake Level (UL) for SIFs in premenopausal women is 150 mg/day.

#### 2.6.2. Estimation of Additional Soy Intake Required to Meet Targets

Soybeans and soy products are among the richest sources of SIFs. For participants whose SIF intake did not meet the applicable SPL, the quantity of additional soybeans and soy products required to reach the target was calculated, and this quantity was subsequently used to estimate the concomitant increases in other nutrients intakes.

### 2.7. Statistical Analysis

All statistical analyses were performed using R (Version 4.5.1). Given the non-normal distribution of SIF intake data, non-parametric tests were applied throughout. For contrast among multiple groups—stratified by age, residential setting (urban vs. rural), PAL, annual household income, educational attainment, occupation, and region—the Kruskal–-Wallis H test was used. Statistically significant was defined as *p* < 0.05. Box plots were generated using Origin 2022 (9.9.0.225) to visualize the distribution of SIF intake relative to applicable SPL across age groups.

## 3. Results

### 3.1. Standardized SIF Intake: Distributional Characteristics and Sociodemographic Variation

Standardized SIF intake among females aged ≥14 years was highly right-skewed, with a mean of 14.77 ± 25.91 mg/standard person-day and a median of 5.32 mg/standard person-day (interquartile range [IQR]: 0.29–19.10 mg/standard person-day).

Standardized SIF intake differed significantly across age groups. Older women generally had higher mean intakes than adolescents and younger adults, with the highest mean intake was observed among women aged ≥65 years, followed by those aged 55–64 years. However, median intake did not increase monotonically with age, suggesting heterogeneity in intake patterns within age strata.

SIF intake also differed significantly across residence type, with rural participants exhibiting higher mean and median intakes than those living in major cities and small and medium-sized cities (SMCs). Intake differed significantly by PAL; participants with light PAL had the highest mean intake, whereas those with vigorous PAL had the lowest. The modest difference in medians values across PAL strata suggests that the observed mean differences were driven primarily by high-intake individuals in the upper-tail of distribution.

Significant differences in SIF intake were detected across annual per capita household income strata and occupational categories. No statistically significant differences were observed across educational levels. Geographic region emerged as a major source of intake variability; participants from the central region recorded the highest mean and median standardized intakes, while those from the western region recorded the lowest (Table 1 and Table S1).

Among age groups, significant differences were observed between the 14~17 and 18~64 years groups (*p* < 0.05), and between the 18~29 and 55~64 years groups (*p* < 0.05), no other pairwise differences reached statistical significance (*p* > 0.05). Across urban–rural settings, large cities differed significantly from both SMCs and rural areas. For PALs, a significant difference was identified between participants with a light PAL and those with moderate-to-vigorous PALs. Although the overall differences in SIF intake across mean annual household income strata reached statistically significant (*p* < 0.05), no individual pairwise contrasts attained significance, suggesting that income-related difference were diffuse rather than concentrated between specific groups. Regarding occupation, students differed significantly from all occupational groups except the “other” category; professionals/technical staff differed significantly from retired/unemployed individuals and administrative staff; and the “other” occupation group differed significantly from professionals/technical staff and commercial service workers. No other occupational comparisons reached statistical significance (*p* > 0.05). All pairwise regional differences were statistically significant.

### 3.2. Dietary Sources of SIF

Across all participants, dried legumes and legume products were the dominant contributors to total SIF intake, accounting for 82.05% of intake. Vegetables and vegetable products ranked the second, contributing 13.01%, followed by condiments (3.25%) and ethnic foods and pastries (1.02%). All other food groups each contributed less than 1%.

Within the dried legumes and legume products, tofu and tofu-derived products represent the largest proportion (67.51%), followed by whole beans (28.60%), with soy-based beverages and others jointly accounting for less than 10%. The relative contributions of different SIF sources were broadly comparable across all sociodemographic subgroups (Tables S2 and S3).

### 3.3. Comparisons of SIF Intake Against the SPL Across Menopausal Status Groups

**Figure 1:**
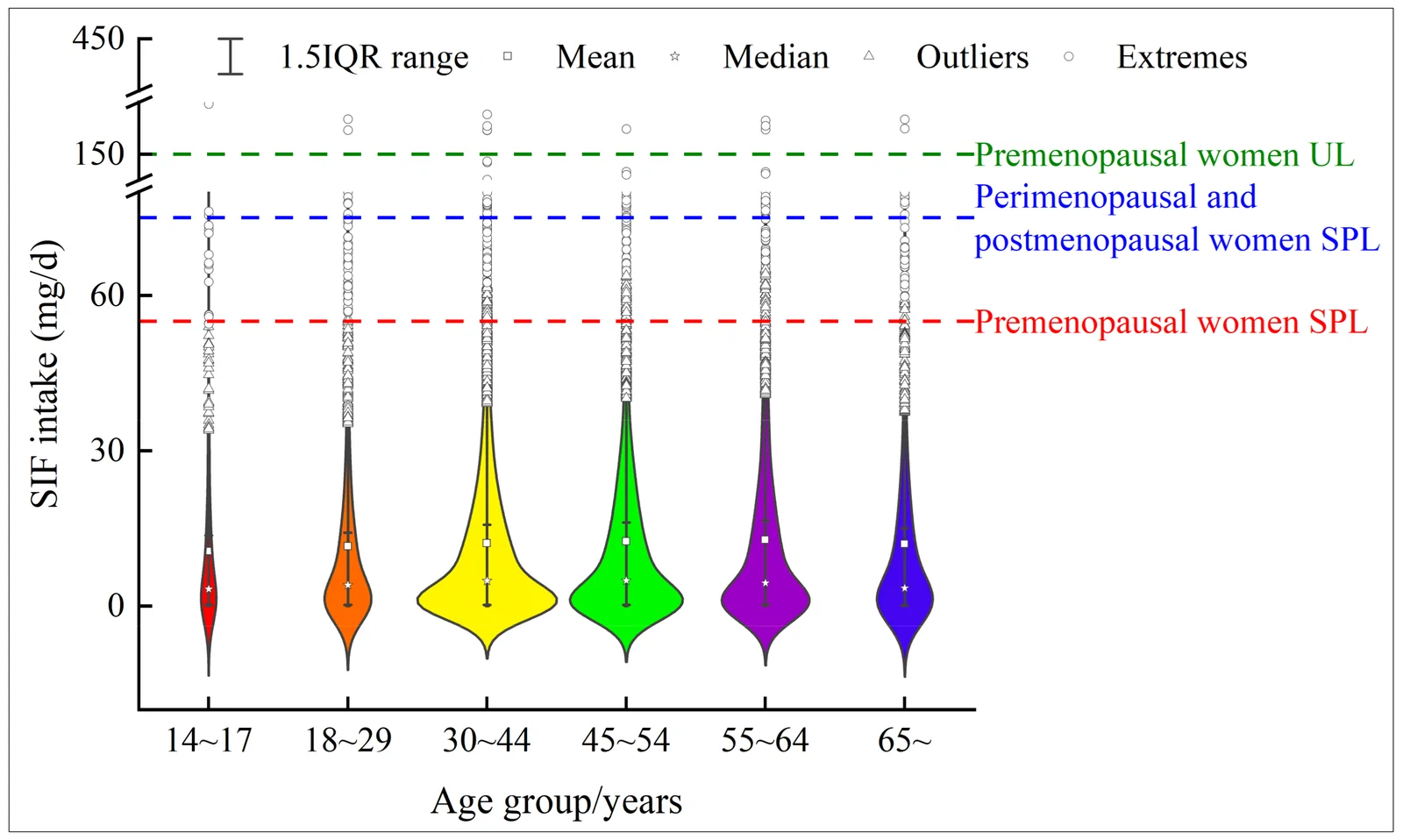
Distribution of Standardized SIF Intake Relative to the SPL Across Age Groups in the Study Population. The *Y*-axis is broken with two breaks to improve visibility of the central distribution while preserving the full range of extreme values.

Across all age groups, the vast majority of participants had SIF intakes below the corresponding SPL, with more than 96% falling short of this population-level target. Substantial gaps between observed intake and the recommended SPL were evident among premenopausal, perimenopausal, and postmenopausal women alike. The proportions of women meeting the SPL were 3.51% for premenopausal women and 2.40% for perimenopausal and postmenopausal women. Among premenopausal women, 0.29% had intakes above the UL of 150 mg/day. The markedly right-skewed distribution of SIF intake indicates that the majority of participants had low intake, whereas a small proportion showed substantially higher values (Figure 1).

It should be noted that these contrasts reflect population-level dietary patterns relative to recommended targets, rather than indicating clinical deficiency or disease risk in individual participants.

### 3.4. Estimated Dietary Augmentation Required to Meet the SPL Through Soy Food Consumption

As illustrated in Figure 1, mean SIF intake across all age groups fell substantially below the SPL, indicating that most participants had considerable room for dietary improvement. Based on an SIF concentration database, soybeans and soy products exhibit the highest SIF content among commonly consumed foods; consequently, increasing their dietary intake represents a feasible strategy for achieving the SPL. Soybeans and soy products include whole beans (e.g., yellow soybeans and green soybeans) as well as fermented and non-fermented processed products. After converting to protein equivalents, the recommended supplemental intake was calculated. Achieving the SPL through increased consumption of soybeans and soy products would concurrently augment intakes of the three major macronutrients as well as key vitamins and minerals (Table 2).

Our recommendations are strictly based on dietary substitutions (e.g., replacing meat or poultry with soy products), and we suggest that future intervention studies should monitor total energy intake and body weight to ensure a net positive effect.

## 4. Discussion

In this cross-sectional study of Chinese women aged ≥14 years, standardized SIF intake was relatively low, with a median of 5.32 mg/standard person-day (IQR: 0.29–19.10 mg/standard person-day). This level was lower than estimates reported in Asian populations, where dietary SIF intake typically ranges from 6.2 to 54.3 mg/day [17,51,52,53,54,55], and substantially higher than intakes reported in Western populations. For example, Seong-Ah Kim et al. used the 24-h dietary recall method to collect food intake data from Korean adults and estimated their isoflavone intake using the Korean Flavonoid Database (KFDB), reporting mean daily intake of an estimated 10.7–39.9 mg/1000 kcal in men and 7.6–23.6 mg/1000 kcal for women [51]. Chisato Nagata et al. administered 169 semi-quantitative FFQs assessing nine common soy foods to 29,079 Japanese adults aged ≥35 years, yielding median SIF intakes ranging from 20.8 mg/day to 65.6 mg/day [52]. By contrast, Makiko Mitsunami et al., using a brief questionnaire, reported a mean SIF intake of only 2.26 mg/day in the general U.S. population [53], while estimates from several European countries were less than 1 mg/day [54,55], underscoring the substantial disparity between Asian and Western dietary patterns.

These inter-country differences in SIF intake are largely attributed to differences in habitual consumption of soybeans and soy products. Furthermore, methodological differences in dietary assessment methods—including the number of food items assessed and the consumption database employed—may contribute to cross-study variability in reported intake levels. In the present study, dried legumes and legume products were the primary contributor, accounting for ~80% of the total. Within this category, tofu and tofu-derived products contributed the largest share (ranging from nearly 52–71%), followed by whole beans. These findings indicate that promoting the consumption of whole beans and tofu products may represent a practical dietary strategy for improving SIF intake among women with suboptimal consumption. This pattern is consistent with prior studies that have identified non-fermented soy products—including soybeans and tofu—as the primary contributors to dietary SIF intake [56,57].

The substantial gap between observed SIF intake and the SPL indicates that the majority of participants across all age groups are below the proposed population-level targets, while only a very small proportion exceeded the UL. Taken together, these findings highlight the potential value of developing differentiated dietary guidance tailored to age, menopausal status, and current intake levels, rather than applying uniform, population-wide recommendations.

As illustrated in Figure 1, women in all age groups in China would need to increase their current SIF intake to meet the SPL. Specifically, premenopausal women would require an additional approximately 40 mg/day of SIF, which corresponds to an increase of nearly 45 g/day of soybeans and soy products, to meet the SPL of 55 mg/day. Perimenopausal and postmenopausal women would require an additional estimated 60 mg/day of SIF, achievable through an increase of about 60 g/day of soy and soy products, to meet the SPL of 75 mg/day.

To achieve the recommended SIF intake, increasing consumption of soybeans and soy products is necessary. However, we emphasize that this increase should be implemented through dietary substitution—replacing a portion of meat, poultry, or other animal-based foods with equivalent amounts of soy products—rather than as an unconditional addition. This approach avoids excess caloric intake (the additional soy needed would contribute ~309–457 kcal/day if simply added) and aligns with overall energy balance goals. Moreover, such substitution offers broader nutritional advantages: per gram of soy products consumed, protein intake more than doubles that from meat or poultry, while calcium, phosphorus, potassium, and magnesium increase by close to 30-, 4-, 5-, and 13-fold, respectively. Monounsaturated and polyunsaturated fatty acids also rise by over two- and three-fold, with a substantially lower cholesterol burden. Although these per-gram increments are modest, habitual substitution over the long term can meaningfully improve overall dietary quality without compromising energy control.

Why is achieving these SIF targets potentially beneficial for women’s health? Previous studies have established SIF as nutritionally important dietary components with potential benefits for women’s health, particularly in the context of menopausal transition. Several systematic reviews and meta-analyses have comprehensively evaluated these health effects. Regarding vasomotor symptoms—a core source of distress during menopausal transition—a meta-analysis of 17 randomized controlled trials reported that SIF intake (median dose 54 mg/day, expressed as aglycone equivalents; duration: 6 weeks to 12 months) significantly reduced hot flash frequency by 20.6% (*p* < 0.00001) and severity by 26.2% (*p* = 0.001) [58]. Nevertheless, some studies have reported no significant improvement in vasomotor symptoms, including hot flashes and night sweats. Current evidence remains limited by variable study quality and substantial heterogeneity across trials, and more rigorous randomized controlled trials are needed to confirm these findings. Of note, an individual’s capacity for endogenous equol production may be a key determinant of SIF efficacy; equol supplementation has been shown to significantly reduce the incidence and/or severity of hot flashes in postmenopausal women who are non-equol producers [59].

With respect to hormone-dependent tumor risk, the relationship between soy isoflavones intake and breast cancer has attracted considerable attention. In this context, large-scale population-based cohort studies suggest that soy and isoflavone intake may be associated with a reduced breast cancer risk. A meta-analysis incorporating nine cohort studies involving 631,498 women found that, within a daily intake range of 19.17 ± 0.5 mg of dietary SIF, each additional 10 mg/day was associated with a 3% reduction in breast cancer risk [60]. The Shanghai Women’s Health Study, a prospective cohort study of 70,578 women aged 40–70 years for a mean of 13.2 years, found that premenopausal women in the highest intake group (55.0 mg/day) had a 46% lower risk of breast cancer compared with those in the lowest intake group (11.1 mg/day) [61]. Similarly, a Japanese cohort study reported that postmenopausal women in the highest intake group (70.6 mg/day) had a 48% lower breast cancer risk than those in the lowest group (18.7 mg/day) [62]. A systematic review and meta-analysis further demonstrated that SIF intake was associated with a 26% reduction in breast cancer recurrence risk (HR = 0.74), with associations particularly pronounced among postmenopausal women and estrogen receptor-positive breast cancer survivors, and the greatest risk reduction observed at a dose of 60 mg/day [63].

From a safety perspective, soy isoflavones may function as selective estrogen receptor modulators, exhibiting tissue-specific bidirectional regulation. A systematic review and meta-analysis of 40 randomized controlled trials involving 3285 participants found that SIF intervention at a median daily dose of 75 mg did not produce significant changes in estrogen-related indicators in postmenopausal women—including endometrial thickness, vaginal maturity index, follicle-stimulating hormone (FSH), and estradiol levels—suggesting that SIF does not exert estrogenic effects in this population and that its mechanism of action differs fundamentally from conventional estrogen therapy [64]. Conversely, in vitro and animal studies indicate that SIF exerts dose-dependent proliferative effects on mammary cells; some researchers argue that adverse effects cannot be completely ruled out at active doses [58]. A meta-analysis of 30 animal models further revealed that dietary isoflavones exert a bidirectional effect in hormone-dependent tumors, simultaneously reducing tumor volume and weight (Hedges’ g = −1.151 and −2.584) while increasing tumor area (Hedges’ g = 1.136). This suggests that caution is warranted when SIF-containing supplements are considered for women with hormone-dependent cancers, particularly at high-dose [65]. Importantly, clinical evidence indicates that SIF do not increase breast cancer-related markers—including breast tissue density and breast cell proliferation—nor do they interfere with the efficacy of tamoxifen and aromatase inhibitors [66]. Overall, current evidence on the safety of SIF remains inconclusive, particularly regarding dose-dependent effects observed in animal models. However, existing clinical data do not indicate significant safety concerns at moderate intake levels, and no evidence suggests interference with standard adjuvant therapies.

Building on this body of evidence, the Dietary Reference Intakes for Chinese Residents (2023) established, for the first time, SPLs for SIF. Specifically, the SPL for premenopausal women is 55 mg/day (to reduce breast cancer risk); while the SPL for perimenopausal and postmenopausal women is 75 mg/day (to reduce breast cancer risk, alleviate perimenopausal syndrome, and improve postmenopausal bone health). The UL for premenopausal women is set at 150 mg/day.

Beyond the issue of quantitative inadequacy, it is critical to recognize that the actual health benefits of meeting the SIF target may vary considerably among Chinese women due to interindividual differences in gut microbiota, which influence the biotransformation and bioavailability of SIF. A key metabolic pathway is the conversion of daidzein to equol—a metabolite with markedly stronger estrogenic, antioxidant, and anti-androgenic activities than its parent compound. However, equol production is strictly dependent on the presence of specific gut bacterial strains, and only an estimated 25–50% of individuals are equol producers (designated as equol-positive) [67,68,69].

Accumulating evidence indicates that equol status modulates the efficacy of SIF interventions. In postmenopausal women, equol supplementation has been shown to significantly reduce the frequency and severity of vasomotor symptoms (VMS), particularly among equol-negative individuals, in whom the therapeutic effect appears to be even more pronounced than in equol-positive women [70,71]. Beyond VMS, daily administration of 10 mg equol three times per day has been reported to alleviate anxiety, depression, and fatigue, as well as to improve muscle stiffness, sweating, and renal function in perimenopausal and postmenopausal women [72,73,74,75].

These findings collectively suggest that equol-producing capacity may serve as a pivotal effect modifier in future intervention studies and personalized dietary guidance. While our current analysis provides population-level estimates of SIF intake adequacy, the translation of these targets into tangible health improvements will likely depend on an individual’s gut microbial ecology, a factor that merits dedicated investigation in future research.

It is important to note, however, that the present study assessed dietary intake rather than clinical outcomes. Accordingly, the findings should be interpreted as evidence of dietary insufficiency relative to current SPL recommendations, rather than as direct evidence that increasing SIF intake would confer protection against specific diseases. The markedly right-skewed distribution of SIF intake observed in this study further highlights substantial heterogeneity in dietary patterns across the population, underscoring the potential value of balanced soy food consumption and targeted nutrition education initiatives.

This study has several limitations that warrant consideration. First, although legumes and legume-derived products constitute a diverse and nutritionally important category of plant-based foods and a key source of high-quality protein in China, available data on their SIF content remain limited in current food composition tables. Moreover, variability in cooking and processing methods may have affected the accuracy of SIF intake estimates. For foods lacking direct SIF-content data, proxy values derived from mean values or compositionally comparable foods were applied, which may have introduced measurement error and potentially led to underestimation or overestimation of true intake. Collectively, these proxy-based estimates contributed less than 5% of the total estimated SIF concentration in our study. Second, although the analyses accounted for multiple demographic and socioeconomic variables—including urban-rural residence, geographic region, household income, and occupation—residual confounding from unmeasured factors cannot be excluded. Third, menopausal status was classified indirectly based on age (<45 years as premenopausal; ≥45 years as perimenopausal/postmenopausal) due to the absence of direct reproductive history in the CFCS. Although this age cutoff is widely adopted in nutritional epidemiological studies when detailed menstrual data are unavailable, it may introduce potential misclassification, particularly for women aged 45–55 years with individual variation in menopause timing. Since the SPL difference and supplementation recommendations are stratified by menopausal status, such misclassification could influence subgroup-specific estimates. However, the uniformly low intake across all age groups and the overwhelming shortfall relative to the SPL (>96% overall) suggest that this limitation does not compromise the primary conclusion of population-level dietary inadequacy. Future studies incorporating validated questionnaires or hormonal biomarkers are warranted to confirm our findings. Fourth, this cross-sectional study design precludes causal inference between SIF intake and health outcomes. Future prospective cohort studies are needed to examine associations between habitual SIF intake and long-term health outcomes, and randomized controlled trials are warranted to clarify the effects of varying SIF intake levels on menopausal symptoms and disease-related biomarkers. Finally, the findings are based on Chinese women, and future studies are needed to determine their applicability to other populations.

## 5. Conclusions

In this cross-sectional study, SIF intake among Chinese women aged ≥14 years was generally low, with the majority of participants falling substantially below the age-specific SPL recommended by the Chinese Dietary Reference Intakes (2023). Dried legumes and legume products—particularly tofu, tofu-derived products, and whole beans—were identified as the primary dietary sources of SIF, followed by vegetables and vegetable products. At the population level, these findings highlight the need for targeted dietary guidance to promote adequate SIF intake among women with low consumption, while also drawing attention to the small subset with intakes above the UL. Nevertheless, as the present study assessed dietary intake rather than health outcomes, these results should be interpreted as evidence of suboptimal dietary patterns rather than as direct evidence that increasing SIF intake would confer clinical protection. Prospective cohort studies and randomized controlled trials are warranted to determine whether improving SIF intake to recommended levels translates into favorable long-term health outcomes in Chinese women.

## Figures and Tables

**Table 1:** Standardized SIF Intake among Females Aged ≥14 Years, 2017–2020 (mg/standard person-day).

Characteristic	*n*	Mean ± SD	P25	P50	P75	*H*	*p*-Value
Overall	20,676	14.77 ± 25.91	0.29	5.32	19.10		
Age (years)						28.15	<0.001
14~17	688	10.53 ± 17.87	0.23	3.41	13.94		
18~29	2113	13.63 ± 24.21	0.26	4.68	16.58		
30~44	6296	14.27 ± 24.50	0.30	5.51	18.62		
45~54	5101	14.84 ± 25.21	0.29	5.69	19.94		
55~64	3959	15.93 ± 27.00	0.29	5.60	20.89		
≥65	2519	16.20 ± 31.42	0.26	4.75	20.79		
Urban and Rural						79.87	<0.001
Major cities	6371	12.48 ± 24.42	0.25	3.76	15.29		
SMCs	6764	15.14 ± 24.45	0.37	5.76	20.41		
Rural	7541	16.38 ± 28.16	0.26	6.32	21.00		
PALs						17.91	<0.001
Light	13,760	15.17 ± 26.91	0.31	5.30	19.50		
Moderate	6008	14.14 ± 24.16	0.25	5.44	18.19		
Vigorous	908	12.95 ± 20.91	0.17	4.72	17.47		
Annual per capita household income						9.55	0.049
Under 10,000 yuan	5115	15.16 ± 27.07	0.24	5.23	19.72		
10,000~39,999 yuan	11,289	14.72 ± 25.56	0.29	5.48	19.21		
40,000~99,999 yuan	2576	15.04 ± 26.66	0.39	5.53	18.60		
Over 100,000 yuan	630	15.51 ± 27.80	0.30	5.15	18.14		
No response	1066	12.41 ± 20.06	0.29	3.89	15.88		
Education level						4.49	0.106
Primary school or below	5228	15.24 ± 26.78	0.23	5.30	19.97		
Middle school	10,919	14.83 ± 25.41	0.29	5.37	19.45		
Associate degree or above	4529	14.10 ± 26.05	0.35	5.23	17.42		
Occupation						47.36	<0.001
Students	978	10.44 ± 17.31	0.24	3.38	13.91		
Retirees or the unemployed	7970	15.05 ± 27.49	0.28	4.91	19.76		
Administrative staff	1806	13.58 ± 23.74	0.32	4.52	16.67		
Professional and technical personnel	2042	15.64 ± 26.01	0.42	6.43	19.96		
Commercial service practitioners	2405	15.14 ± 25.58	0.34	6.23	19.35		
Manual workers	3880	16.11 ± 27.22	0.25	6.04	20.89		
Others	1595	12.48 ± 20.63	0.24	4.53	16.69		
Area						399.71	<0.001
Eastern	9878	13.43 ± 25.40	0.27	3.99	16.77		
Central	5000	21.01 ± 31.14	0.53	10.53	27.19		
Western	5798	11.68 ± 20.29	0.21	3.91	15.62		

**Table 2:** Estimated Dietary Augmentation of Soybean and soy products Required to meet the SIF, with associated changes in nutrient intake.

Increments in Consumption	Age Group/Years					
	14~17	18~29	30~44	45~54	55~64	65~
SIF reaches SPL (mg/d)	44.47	41.37	40.73	60.16	59.07	58.80
Soybeans and soy products (g/d)	47.81	44.48	43.79	64.68	63.50	63.21
Energy (kcal/d)	338.08	314.51	309.64	457.36	449.07	447.02
Protein (g/d)	17.24	16.03	15.79	23.32	22.89	22.79
Fat (g/d)	24.92	23.19	22.83	33.72	33.11	32.95
Carbohydrate (g/d)	12.92	12.02	11.84	17.48	17.17	17.09
Dietary fiber (g/d)	1.53	1.42	1.40	2.06	2.03	2.02
Cholesterol (mg/d)	0.12	0.11	0.11	0.16	0.16	0.16
Saturated fats (g/d)	3.33	3.10	3.05	4.51	4.43	4.41
Monounsaturated fats (g/d)	5.01	4.66	4.59	6.78	6.65	6.62
Polyunsaturated fatty acids (g/d)	11.68	10.86	10.70	15.80	15.51	15.44
Vitamin A (μg RAE/d) *	11.28	10.50	10.33	15.26	14.99	14.92
Vitamin E (mg/d)	25.25	23.49	23.13	34.16	33.55	33.39
Ca (mg/d)	161.60	150.33	148.01	218.61	214.65	213.67
P (mg/d)	226.72	210.92	207.66	306.72	301.16	299.78
K (mg/d)	454.21	422.55	416.01	614.47	603.34	600.58
Mg (mg/d)	110.51	102.81	101.22	149.50	146.79	146.12

* μg RAE, Retinol Activity Equivalents.

## Data Availability

Data sharing is not applicable to this study, according to the China National Center for Food Safety Risk Assessment.

## Supplementary Materials

The following supporting information can be downloaded at: https://www.mdpi.com/article/10.3390/nu18162696/s1, Table S1: Standardized SIF Intake among Females Aged ≥14 Years, 2017–2020 (mg/standard person-day); Table S2: Contribution of Common Food Groups to Total SIF Intake (%); Table S3: SIF Contribution Rates for the Legumes and Legume Products Subcategory (%).

## Abbreviations

The following abbreviations are used in this manuscript:

SIF	Soy isoflavones
ERT	Estrogen replacement therapy
SERM	Selective estrogen receptor modulator
SPL	Specific proposed level
kcal	kilocalorie
PAL	Physical activity level
STRAW	Stages of reproductive aging workshop
EER	Estimated energy requirement
DRIs	Dietary reference intakes
UL	Tolerable upper intake level
