# Supplementary material for: Assessment of Dietary Soy Isoflavones Intake Among Women Aged 14 Years and Older in China: A Population-Based Cross-Sectional Study
Source: Nutrients. 2026 Aug 18;18(16):2696. doi: 10.3390/nu18162696 (PMC13517065; doi:10.3390/nu18162696)
Supplement: Supplementary file 1 [file nutrients-18-02696-s001.zip › nutrients-4395293-supplementary.pdf]

Table S1. Standardized SIF Intake among Females Aged ⩾14 Years, 2017-2020 (mg/standard person-day).

Characteristic	n	$\overline{X} \pm SD$	P2.5	P5	P25	P50	P75	P95	P97.5	*H*	*p*-Value
Overall	20676	14.77 ± 25.91	0.02	0.05	0.29	5.32	19.10	59.65	84.87		
Age(years)										28.15	<0.001
14~17	688	10.53 ± 17.87	0.02	0.03	0.23	3.41	13.94	44.78	65.50		
18~29	2113	13.63 ± 24.21	0.02	0.05	0.26	4.68	16.58	56.37	77.93		
30~44	6296	14.27 ± 24.50	0.03	0.05	0.30	5.51	18.62	56.54	79.90		
45~54	5101	14.84 ± 25.21	0.03	0.05	0.29	5.69	19.94	58.07	87.70		
55~64	3959	15.93 ± 27.00	0.03	0.05	0.29	5.60	20.89	64.77	90.57		
⩾65	2519	16.20 ± 31.42	0.02	0.04	0.26	4.75	20.79	65.89	96.86		
Urban and Rural										79.87	<0.001
Major cities	6371	12.48 ± 24.42	0.03	0.05	0.25	3.76	15.29	48.77	69.71		
SMCs	6764	15.14 ± 24.45	0.03	0.06	0.37	5.76	20.41	62.04	85.51		
Rural	7541	16.38 ± 28.16	0.02	0.04	0.26	6.32	21.00	65.35	94.79		
PALs										17.91	<0.001
Light	13760	15.17 ± 26.91	0.03	0.05	0.31	5.30	19.50	62.07	89.10		
Moderate	6008	14.14 ± 24.16	0.02	0.04	0.25	5.44	18.19	54.74	76.58		
Vigorous	908	12.95 ± 20.91	0.01	0.03	0.17	4.72	17.47	55.24	78.90		
Annual per capita household income										9.55	0.049
Under 10,000 yuan	5115	15.16 ± 27.07	0.02	0.04	0.24	5.23	19.72	59.74	87.00		
10000~39999 yuan	11289	14.72 ± 25.56	0.03	0.05	0.29	5.48	19.21	59.18	82.32		
40000~99999 yuan	2576	15.04 ± 26.66	0.04	0.07	0.39	5.53	18.60	63.07	91.52		
Over 100,000 yuan	630	15.51 ± 27.80	0.03	0.06	0.30	5.15	18.14	60.76	101.32		
No response	1066	12.41 ± 20.06	0.03	0.05	0.29	3.89	15.88	51.44	68.01		
Education level										4.49	0.106
Primary school or below	5228	15.24 ± 26.78	0.02	0.04	0.23	5.30	19.97	61.93	91.10		
Middle school	10919	14.83 ± 25.41	0.02	0.05	0.29	5.37	19.45	59.57	82.20		
Associate degree or above	4529	14.10 ± 26.05	0.03	0.06	0.35	5.23	17.42	55.28	81.63		
Occupation										47.36	<0.001
Students	978	10.44 ± 17.31	0.02	0.04	0.24	3.38	13.91	44.6	59.76		
Retirees or the unemployed	7970	15.05 ± 27.49	0.02	0.05	0.28	4.91	19.76	59.84	85.89		
Administrative staff	1806	13.58 ± 23.74	0.03	0.05	0.32	4.52	16.67	55.31	81.41		
Professional and technical personnel	2042	15.64 ± 26.01	0.05	0.07	0.42	6.43	19.96	65.32	91.25		
Commercial service practitioners	2405	15.14 ± 25.58	0.03	0.06	0.34	6.23	19.35	58.60	86.72		
Manual workers	3880	16.11 ± 27.22	0.02	0.04	0.25	6.04	20.89	68.11	96.38		
Others	1595	12.48 ± 20.63	0.03	0.05	0.24	4.53	16.69	51.07	66.63		
Area										399.71	<0.001
Eastern	9878	13.43 ± 25.40	0.03	0.06	0.27	3.99	16.77	53.96	75.14		
Central	5000	21.01 ± 31.14	0.02	0.05	0.53	10.53	27.19	82.17	116.29		
Western	5798	11.68 ± 20.29	0.02	0.03	0.21	3.91	15.62	46.14	64.74		

Table S2. Contribution of Common Food Groups to Total SIF Intake (%).

Characteristic	Dried Legumes and Legume Products	Vegetables and Vegetable Products	Condiments	Ethnic foods and Pastries	Cereals and Cereal Products	Fruits and Fruit Products	Tubers, Starches and Products	Nuts and Seeds	Fungi and Algae	Milk and Milk Products	Fast foods
Overall	82.05	13.01	3.25	1.02	0.27	0.21	0.13	0.03	0.02	0.01	0.00
Age(years)											
14~17	81.58	13.03	3.78	0.93	0.30	0.23	0.06	0.01	0.03	0.04	0.00
18~29	83.56	11.81	3.12	0.91	0.24	0.22	0.10	0.02	0.02	0.00	0.00
30~44	82.78	12.50	3.09	0.97	0.27	0.22	0.11	0.02	0.03	0.01	0.00
45~54	81.44	13.26	3.40	1.20	0.29	0.22	0.13	0.03	0.02	0.01	0.00
55~64	79.60	15.33	3.34	1.03	0.29	0.19	0.16	0.03	0.02	0.00	0.00
≥65	84.53	10.81	3.17	0.84	0.24	0.16	0.20	0.03	0.02	0.00	0.00
Urban and Rural											
Major cities	83.02	11.80	3.47	0.92	0.30	0.31	0.13	0.03	0.03	0.00	0.00
SMCs	82.05	12.39	3.42	1.43	0.27	0.24	0.14	0.04	0.03	0.00	0.00
Rural	81.46	14.24	2.99	0.75	0.25	0.12	0.13	0.01	0.02	0.02	0.00
PALs											
Light	82.45	12.38	3.31	1.13	0.28	0.25	0.14	0.03	0.02	0.01	0.00
Moderate	81.31	14.27	3.09	0.74	0.26	0.15	0.14	0.02	0.02	0.00	0.00
Vigorous	81.73	12.94	3.48	1.38	0.23	0.10	0.10	0.02	0.01	0.00	0.00
Annual per capita household income											
Under 10,000 yuan	82.44	12.94	2.89	1.13	0.26	0.15	0.16	0.02	0.02	0.00	0.00
10000~39999 yuan	81.98	13.03	3.33	1.03	0.26	0.21	0.12	0.02	0.02	0.01	0.00
40000~99999 yuan	80.65	13.56	3.65	1.27	0.34	0.33	0.15	0.03	0.03	0.00	0.00
Over 100,000 yuan	83.70	12.46	2.98	0.10	0.25	0.33	0.12	0.04	0.03	0.00	0.00
No response	83.55	11.95	3.58	0.14	0.35	0.19	0.18	0.04	0.03	0.00	0.00
Education level											

Characteristic	Dried Legumes and Legume Products	Vegetables and Vegetable Products	Cond iment s	Ethnic foods and Pastries	Cereals and Cereal Products	Fruits and Fruit Products	Tubers, Starches and Products	Nuts and Seeds	Fungi and Algae	Milk and Milk Products	Fast food s
Primary school or below	81.12	14.71	2.99	0.58	0.26	0.13	0.17	0.02	0.02	0.01	0.00
Middle school	81.58	13.25	3.37	1.13	0.26	0.22	0.13	0.03	0.02	0.01	0.00
Associate degree or above	84.44	10.23	3.28	1.29	0.31	0.29	0.11	0.03	0.03	0.00	0.00
Occupation											
Students	80.95	13.32	3.95	1.06	0.33	0.26	0.06	0.02	0.03	0.03	0.00
Retirees or the unemployed	82.04	13.21	3.02	0.99	0.31	0.22	0.15	0.03	0.02	0.01	0.00
Administrative staff	83.24	10.81	3.67	1.43	0.35	0.32	0.11	0.03	0.03	0.01	0.00
Professional and technical personnel	84.50	10.37	3.59	0.82	0.25	0.27	0.13	0.03	0.03	0.00	0.00
Commercial service practitioners	84.33	10.68	3.15	1.21	0.26	0.22	0.10	0.03	0.02	0.00	0.00
Manual workers	80.56	14.81	3.21	0.91	0.20	0.11	0.17	0.01	0.02	0.00	0.00
Others	78.19	16.80	3.47	0.99	0.21	0.19	0.11	0.02	0.02	0.00	0.00
Area											
Eastern	80.46	13.42	3.87	1.43	0.32	0.31	0.13	0.03	0.03	0.00	0.00
Central	85.90	10.66	2.16	0.82	0.19	0.11	0.13	0.01	0.02	0.00	0.00
Western	79.27	15.74	3.73	0.54	0.31	0.17	0.16	0.04	0.02	0.03	0.00

Table S3. SIF Contribution Rates for the Legumes and Legume Products Subcategory (%).

Characteristic	Tofu and tofu-derived Products	Whole beans	Soy-based beverages	Others *
Overall	67.51	28.60	3.48	0.41
Age(years)				
14~17	68.27	26.06	5.67	1.01
18~29	65.32	29.57	4.71	0.39
30~44	68.88	27.69	3.38	0.05
45~54	67.49	29.04	2.68	0.79
55~64	69.27	27.36	2.86	0.51
≥65	62.96	31.94	4.85	0.25
Urban and Rural				
Major cities	65.78	30.54	3.56	0.12
SMCs	71.49	25.00	3.32	0.18
Rural	65.38	30.28	3.56	0.78
PALs				
Light	67.57	28.62	3.52	0.28
Moderate	67.13	29.08	3.32	0.47
Vigorous	69.02	25.29	4.01	1.68
Annual per capita household income				
Under 10,000 yuan	66.96	28.81	3.80	0.43
10000~39999 yuan	69.19	27.22	3.14	0.45
40000~99999 yuan	62.69	32.26	4.79	0.26
Over 100,000 yuan	57.62	40.54	1.84	0.00
No response	70.55	25.55	3.35	0.55
Education level				
Primary school or below	66.13	29.93	3.36	0.57
Middle school	68.97	27.38	3.19	0.45
Associate degree or above	65.53	30.02	4.35	0.11
Occupation				
Students	68.33	25.25	5.66	0.76
Retirees or the unemployed	67.72	29.47	2.61	0.20
Administrative staff	68.69	28.03	3.15	0.13
Professional and technical personnel	66.54	29.09	4.34	0.03
Commercial service practitioners	67.64	27.81	3.84	0.71
Manual workers	66.15	29.02	4.21	0.62
Others	70.27	25.36	3.17	1.20
Area				
Eastern	65.63	30.57	3.40	0.40
Central	68.56	26.89	4.16	0.39
Western	69.36	27.62	2.55	0.46

* Other categories include bean-based fillings, plant-based meat alternatives, and pastries.
